# Clinical Significance and Next-Generation Sequencing of Chinese Pulmonary Sarcomatoid Carcinoma

**DOI:** 10.1038/s41598-017-04296-2

**Published:** 2017-06-21

**Authors:** Xin Li, Dan Wang, Qingchun Zhao, Dian Ren, Fan Ren, Gang Chen, Hongyu Liu, Jun Chen

**Affiliations:** Tianjin Key Laboratory of Lung Cancer Metastasis and Tumor Microenvironment, Tianjin Lung Cancer Institute; Department of Lung Cancer Surgery, Tianjin Medical University General Hospital; Heping District, Tianjin, 300052 China

## Abstract

Pulmonary Sarcomatoid Carcinoma (PSC) constitutes a heterogeneous group of non-small-cell lung carcinomas (NSCLCs) with a poor prognosis. In this study, a group of 7 patients with PSC was studied. Microscope analysis of all 7 cases revealed a pleomorphic carcinoma subtype. Moreover, 5 cases (71.4%) were composed entirely of malignant sarcomatoid-like elements, and 2 cases (28.6%) were composed of malignant sarcomatoid-like elements and at least 10% adenocarcinoma-like elements. Immunohistochemically, the PSC components of all 7 cases were positive for vimentin and cytokeratins, including cytokeratin (CK) and cytokeratin 7 (CK7). Next-Generation Sequencing (NGS) was performed, and a total of 136 putative somatic variants and one gene fusion were identified, of which 16 variants were considered hot spot mutations, including the genes EGFR, EML4-ALK, MET, BRAF, PIK3CA, and TP53. Of these hot spot mutations, one sample expressing an EML4-ALK fusion was further confirmed by Ventana IHC, and one sample containing an EGFR exon 19 deletion was also confirmed. The NGS results imply that TP53 mutations occur often in PSCs and that EML4-ALK fusion events and EGFR exon deletions also occur in these rare tumors. Molecular targeted therapy may be a useful treatment strategy for these rare lung tumors.

## Introduction

Pulmonary sarcomatoid carcinomas (PSCs) account for less than 0.4% of all lung tumors. They constitute a heterogeneous group of non-small cell lung carcinomas that contain a sarcoma or a sarcoma-like (spindle and/or giant cell) component. According to the 2004 World Health Organization (WHO) classification of lung tumors, PSC can be divided to five subtypes: pleomorphic carcinoma, spindle cell carcinoma, giant cell carcinoma, carcinosarcoma, and pulmonary blastoma^1^. PSC patients show a poor prognosis, with an overall 5-year survival rate of 25%, compared with 45% for other types of non-small cell lung carcinomas, even with aggressive surgical treatment and postoperative chemotherapy. In a Surveillance Epidemiology and End Results database analysis, PSC patients showed a significantly shorter overall survival (OS) (hazard ratio [HR] = 1.60; confidence interval [CI] 95%: 1.35–3.06; p = 0.004) compared with that of other early-stage NSCLC patients^2^. The efficacy of chemotherapy for PSCs is unsatisfactory, with only 31% of patients showing disease control, 16.5% of patients showing a partial response (PR), and 14.5% showing stable disease. Moreover, 69% of PSC patients showed disease progression after a median number of three cycles of chemotherapy^3^. The median progression-free survival (PFS) in PSC patients is 2.0 months (CI 95%: 1.8–2.3), and the median OS is 7.0 months (CI 95%: 4.9–9.0), even in patients treated with platinum-based chemotherapy. These survival rates are worse than those for other NSCLC histological subtypes. In an analysis of three randomized studies that included 984 patients with NSCLC of histological subtypes other than PSCs, the progression rate to first-line chemotherapy was 38%, the median PFS was 4.3 months, and the median OS was 8.9 months^4^. Chemotherapy is associated with significant side effects in all NSCLC patients, including PSC patients, due to the lack of specificity. Recently, the development of targeted therapy drugs for NSCLC has garnered increased attention, and remarkable successes have been reported in several NSCLC studies^5, 6^. NSCLC patients who carry an EGFR activating mutation and a fusion protein between the N-terminal portion of the echinoderm microtubule-associated protein-like 4 protein and the intracellular signaling portion of the anaplastic lymphoma kinase tyrosine kinase receptor (EML4-ALK) are sensitive to tyrosine kinase inhibitors (TKIs). However, few studies on the molecular analysis of PSCs regarding the frequency of these mutations are available^7–9^. Thus, more studies on the molecular status of PSC are needed for a deeper understanding of this disease and for guiding therapy decisions. Therefore, we performed next-generation sequencing (NGS) on our collection of clinical and pathological characteristics in 7 Chinese PSC patients to identify potential driver genes and to identify potentially effective drug targets.

## Materials and Methods

### Ethical approval

This study was conducted in accordance with the standards of the Declaration of Helsinki for medical research involving human subjects. All subjects provided informed consent, and the study protocol was approved by the clinical research ethical review board at Tianjin Medical University General Hospital.

For specimens older than 2 years, we were unable to conduct high-throughput sequencing because of DNA fragmentation; therefore, only 7 PSC samples collected between 2014 and 2015 were included in our study. The patients were admitted to the Department of Lung Cancer Surgery at Tianjin Medical University General Hospital. All PSC cases were reviewed according to the WHO criteria and were staged according to the American Joint Committee staging manual (seventh edition) criteria^10^. Sarcomatoid components include the presence of spindle cells, giant cells, and mixed cell types. Spindle cell components are composed of fusiform malignant cells that are reactive to epithelial marker antibodies such as cytokeratin (CK), cytokeratin 7 (CK7), carcinoembryonic antigen (CEA), and epithelial membrane antigen (EMA). Giant cell components are composed of giant tumor cells with abundant cytoplasm and multiple nuclei or a single large pleomorphic nucleus. The mixed type exhibits a mixture of spindle cells and giant cells, each of which comprises at least 10% of the sarcomatoid areas. Cases in which neoadjuvant radiotherapy or chemotherapy had been performed were excluded from the analysis.

Four-micrometer-thick sections were prepared from paraffin-embedded tissue blocks using 10% methanol solution as a fixative. The sections were stained with hematoxylin and eosin (H&E) or with periodic acid–Schiff (PAS), alcian blue, or mucicarmine stains. Immunohistochemical staining was performed using the avidin-biotin-peroxidase complex method.

In this study, NGS was performed on DNA isolated from formalin-fixed paraffin-embedded (FFPE) tumor tissue after operation or biopsy using the TruSeq Amplicon–Cancer 295 gene panel (Guangzhou Burning Rock Biotechnology Inc. China). DNA was extracted using the FFPE Plus LEV DNA Purification Kit (Promega, Madison, WI) and a Maxwell 16 instrument (Promega). DNA quality and

quantity were assessed using a Nanodrop and Qubit (Thermo Fisher Scientific, Waltham, MA). All genes are shown in Complementary Table 1. Sample analysis included a DNA quality control analysis followed by the generation, quantification, and normalization of the libraries, which were sequenced using a MiSeq Desktop Sequencer instrument (Burning Rock Biotechnology). The data were analyzed using the AmpliconDS protocol and VariantStudio (Burning Rock Biotechnology) and Integrated Genomics Viewer 2.3 software (https://www.broadinstitute.org/igv/). The dual strand approach implemented in this panel allows for the elimination of the frequent erroneous mutation calls in FFPE samples owing to the fixation-derived deamination of DNA. Somatic variants were identified by selecting mutations with no reported minor allele frequency in the germline mutation repositories according to the Variant Studio software. This approach was verified by subjecting the matching non-cancer tissues of 5 surgical PSCs to deep sequencing. After the NGS test, the ALK protein was evaluated using a monoclonal rabbit antibody (Ventana D5F3, ROCHE, CH) and a benchmark system. ALK was considered positive when at least 10% of tumor cells stained with an intensity ≥ 2. The H2228 cell line was used as an external positive control.

**Table 1:** Clinical features of the 7 patients with Pulmonary Sarcomatoid Carcinoma.

No.	Sex	Age	Smoking status	Source of diagnostic material	Tumor Location	Pleural invasion	Subtypes	Lymph node involvement	Tumor Size	Stage	Survival
1	F	57	N	S	RLL	Yes	Variant 2	Yes	4.5 cm	IIIa	N
2	F	64	N	S	LLL	No	Variant 2	No	3 cm	Ib	N
3	M	60	Y	B	RUL	NK	Variant 2	NK	5.5 cm	IV	N
4	F	38	N	B	RLL	NK	Variant 2	NK	7 cm	IV	N
5	M	65	N	S	RML	No	Variant 1	No	3 cm	Ib	Y
6	M	73	N	S	RLL	Yes	Variant 1	No	12 cm	IIb	Y
7	M	56	Y	S	RUL	Yes	Variant 2	No	13 cm	IIIa	Y

M: male, F: female, NK: not known, RUL: right upper lobe, LUL: left upper lobe, RLL: right lower lobe, RML: right middle lobe, L: left; B: biopsy; S: surgical resection.

## Results

### Clinical Features of the Study Cohort

The clinical data of the 7 cases are summarized in Table 1. The patients included 4 men and 3 women (male to female ratio, 1.3:1) ranging in age from 38–73 years (mean 59 years). The tumor size ranged from 3–13 centimeters (mean, 6.9 centimeters). In all 7 cases, 2 patients (28.6%, 2/7) had a history of smoking, 6 patients (85.7%, 6/7) presented with tumors in the right lung, and 1 patient presented with a tumor in the left lung. In the pathologic staging, 2 patients (28.6%, 2/7) had clinical Stage Ib disease, 1 patient (14.3%, 1/7) had Stage IIb disease, 2 patients (28.6%, 2/7) had Stage IIIa disease, and 2 patients (28.6%, 2/7) had Stage IV disease. Lobectomy and systematic mediastinal lymphadenectomy were performed in five stage I to stage III patients. Two stage IV patients received only a pulmonary biopsy. In the 5 patients who accepted surgical treatment, only 1 patient (case 1) had lymph node involvement in groups 7 and 11. Case 3 and case 4 had confirmed distant organ metastasis through preoperative systematic imaging; however, they only accepted a pulmonary biopsy. Thus, we were unable to confirm their lymph node status.

### Microscope Findings

According to the WHO, PSC has five histologic subtypes: pleomorphic carcinoma, spindle cell carcinoma, giant cell carcinoma, carcinosarcoma, and pulmonary blastoma. Carcinosarcoma and pulmonary blastoma are relatively rare compared with the other 3 subtypes. Pleomorphic carcinoma has the highest incidence of the 5 subtypes^11^ and consists of two variants: variant 1 is defined as a poorly differentiated, non-small cell carcinoma, adenocarcinoma, squamous cell carcinoma, or large cell carcinoma, admixed with at least 10% malignant sarcomatoid components (spindle cells and/or giant cells). Variant 2 is defined as a carcinoma composed only of malignant sarcomatoid components (spindle cells and giant cells). According to the WHO, spindle cells vary morphologically from epithelioid cells and are strikingly spindled and arranged in haphazard fascicles or in a storiform pattern. Malignant giant cells are discohesive; uninucleated or multinucleated; have moderate to abundant, dense, eosinophilic cytoplasm; often show emperipolesis by polymorphonuclear leukocytes or lymphocytes; and are typically anaplastic with many bizarre forms. Tumor cells are embedded in a fibrous or often myxoid stroma. Spindle cell carcinoma is defined as a non-small cell carcinoma composed of only malignant spindle cells. Giant cell carcinoma is defined as a non-small cell carcinoma composed of only malignant giant cells.

**Figure 1:**
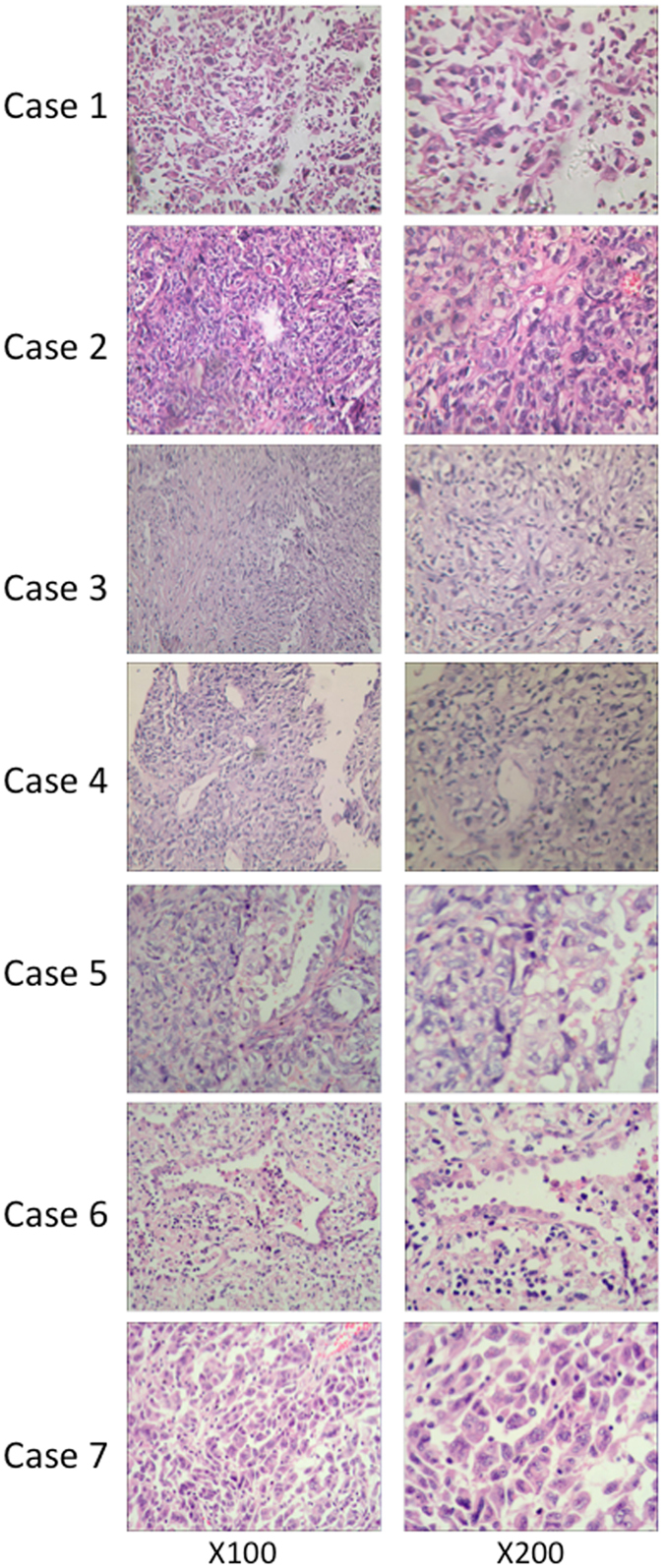
Pathological characteristics demonstrated by H&E staining. Cases 1, 2, 3, 4, and 7 were composed entirely of malignant sarcomatoid cells (spindle cells and malignant cells) and showed poorly differentiated bizarre giant cells that were distributed evenly among abundant spindle-shaped cells. In cases 5 and 6, we observed an adenoid structure among the malignant sarcomatoid components.

In our study, all patients were diagnosed with pleomorphic carcinoma, as shown in Fig. 1. Five cases (71.4%) were composed entirely of malignant sarcomatoid components, including cases 1, 2, 3, 4, and 7. The remaining 2 cases (28.6%) were composed of malignant sarcomatoid components admixed with at least 10% adenocarcinoma components, including cases 5 and 6. Usually, sarcomatoid components are further subdivided into 3 types: spindle cell type, giant cell type, and mixed type. All of our cases were mixed type. We observed poorly differentiated bizarre giant cells distributed evenly among abundant spindle-shaped cells. In cases 5 and 6, we also observed mesenchyme with loose myxoid areas and malignant giant cells showing loose adhesion in a polygonal shape with single-cores or multi-cores.

### Immunohistochemical Findings

**Figure 2:**
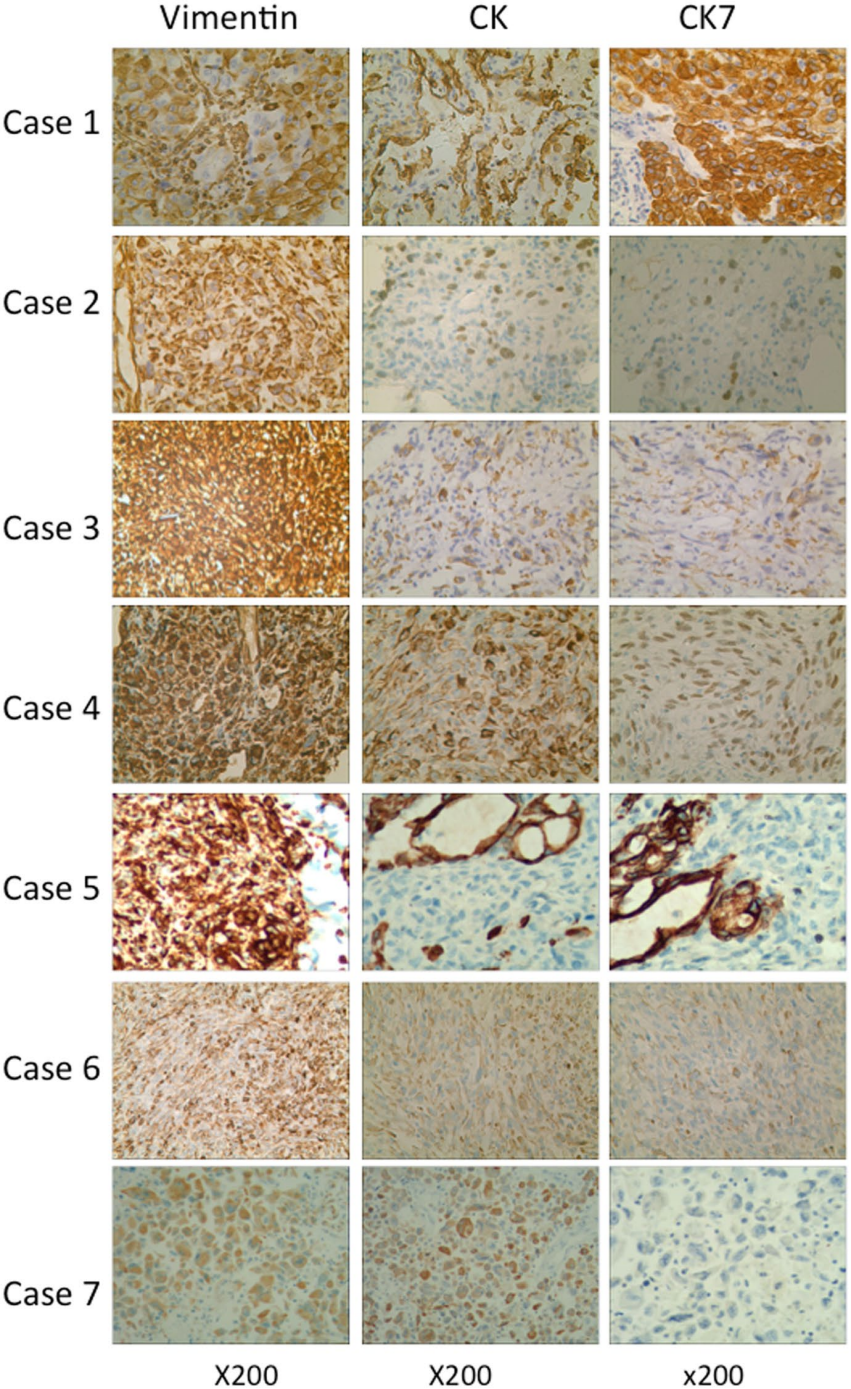
Immunohistochemical characteristics of our study. All 7 cases were reactive to vimentin and cytokeratin immunostaining, showing diffuse staining in the cytoplasm. Cases 1–6 was positive for CK7 staining, whereas case 7 was negative for CK7 staining.

The immunohistochemical results of all 7 cases are shown in Table 2. All 7 cases (100%) stained positive for vimentin, and all 7 cases (100%) stained positive for CK, with partial reactivity in case 2 and case 4. Six cases were reactive to CK7 (85.7%); no staining was observed in case 7, partial reactivity was observed in cases 2 and 4, and strong reactivity was observed in cases 1, 3, 5, and 6. Two of seven cases were reactive to EMA (28.6%), 3 of 7 cases were reactive to P63 (42.9%), 1 of 7 cases was reactive to CK5/6 (14.3%), 3 of 7 cases were reactive to TTF-1 (42.9%), and all cases were negative for CgA. The vimentin, CK, and CK7 immunohistochemical results are shown in Fig. 2.

**Table 2:** Immunohistochemical results for the 7 patients with Pulmonary Sarcomatoid Carcinoma.

No.	CK	CK7	Vimentin	EMA	P63	CK5/6	TTF-1	CgA
1	+	+	+	+	−	−	+	−
2	+	+	+	−	+	+	−	−
3	+	+	+	−	+	−	−	−
4	+	+	+	−	+	−	+	−
5	+	+	+	−	−	−	−	−
6	+	+	+	+	−	−	+	−
7	+	+	+	−	−	−	−	−

CK: cytokeratin, CK7: cytokeratin 7, EMA: epithelial membrane antigen, CK5/6: cytokeratin 5/6, TTF-1: thyroid transcription factor-1, CgA: chromogranin A.

### NGS Findings

**Figure 3:**
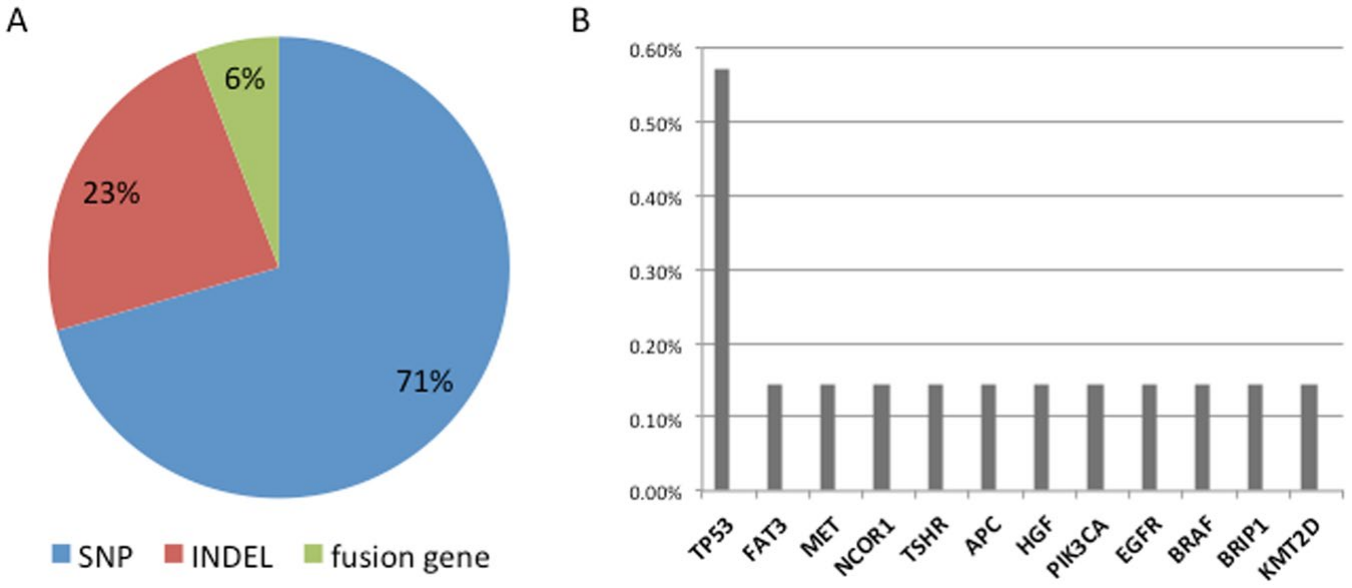
(**A**) Mutation types according to the type of nucleotide substitution and the variant consequence. (**B**) Somatic mutation events detected in PSC.

The NGS results are shown in Table 3. For the depth of sequencing, 88.9% of target regions were covered >200-fold, 55.8% of target regions were covered >500-fold, and 25.9% of target regions were covered >1000-fold. Of the 136 total putative somatic variants, 16 variants were considered hot spot mutations. In addition, one gene fusion was identified. Most of these mutations were single nucleotide polymorphisms (SNPs, 71%), followed by insertions or deletions (INDELs, 23%), and gene fusion (6%), as shown in Fig. 3A. All 7 PSCs showed at least one mutation and 6 patients (85.7%) showed multiple mutations in a single gene (up to 5 different mutations).

**Table 3:** The NGS results of our cohort.

ID	Sex	chr:posi	ref > alt	type	Gene name	Amino acid change	Codon change	Effect	Impact	Transcript Id	Cosmic id	Cosmic allele
1	F	11:92087139	C > T	SNP	FAT3	L621F	CTT/TTT	missense_variant	MODERATE	ENST00000298047	COSMIC1206618	p.L621F
		7:116339746	C > T	SNP	MET	S203F	TCT/TTT	missense_variant	MODERATE	ENST00000318493	COSMIC282765	p.S203Y
		17:16049843	C > T	SNP	NCOR1	R310H	CGT/CAT	missense_variant	MODERATE	ENST00000268712	COSMIC1216870	p.R310H
		14:81534648	A > T	SNP	TSHR	Y98F	TAC/TTC	missense_variant	MODERATE	ENST00000541158	COSMIC553821	p.Y98C
		5:112174682	CAAG > C	INDEL	APC	QE1131Q	CAAGAA/CAA	inframe_deletion	MODERATE	ENST00000508376	COSMIC13868	p.Q1131*
2	F	7:81336664	C > G	SNP	HGF	G520R	GGA/CGA	missense_variant	MODERATE	ENST00000222390	COSMIC79203	p.G520*
		17:7577551	C > A	SNP	TP53	G244C	GGC/TGC	missense_variant	MODERATE	ENST00000269305	COSMIC10941	p.G244S
3	M	3:178952088	A > G	SNP	PIK3CA	H1048R	CAT/CGT	missense_variant	MODERATE	ENST00000263967	COSMIC36289	p.H1048R
		3:178952085	A > G	SNP	PIK3CA	H1047R	CAT/CGT	missense_variant	MODERATE	ENST00000263967	COSMIC94987	p.H1047L
		17:7578416	C > A	SNP	TP53	V172F	GTT/TTT	missense_variant	MODERATE	ENST00000269305	COSMIC43955	p.V172I
4	F	17:7577085	C > T	SNP	TP53	E285K	GAG/AAG	missense_variant	MODERATE	ENST00000269305	COSMIC44388	p.E285*
5	M	7:55242464	AGGAATTAA-GAGAAGC > A	INDEL	EGFR	KELREA745K	AAGGAATTAA-GAGAAGCA/AAA	disruptive_inframe_deletion	MODERATE	ENST00000275493	COSMIC51504	p.K745_E746insIPVAIK
		17:7579419	A > AG	INDEL	TP53	P89P	CCC/CCCC	frameshift_variant	HIGH	ENST00000269305	COSMIC1735386	p.S90T
6	M	7:140481402	C > G	SNP	BRAF	G469A	GGA/GCA	missense_variant	MODERATE	ENST00000288602	COSMIC460	p.G469A
7	M	17:59876521	T > C	SNP	BRIP1	N427S	AAC/AGC	missense_variant	MODERATE	ENST00000259008	COSMIC3742356	p.N427S
		12:49426753	AGTTGCT > A	INDEL	KMT2D	QQL3910L	CAGCAACTT/CTT	disruptive_inframe_deletion	MODERATE	ENST00000301067	COSMIC1190862	p.Q3910_L3912 > H

chr: chromosome, posi: position, ref: reference genome, alt: gene alteration.

**Figure 4:**
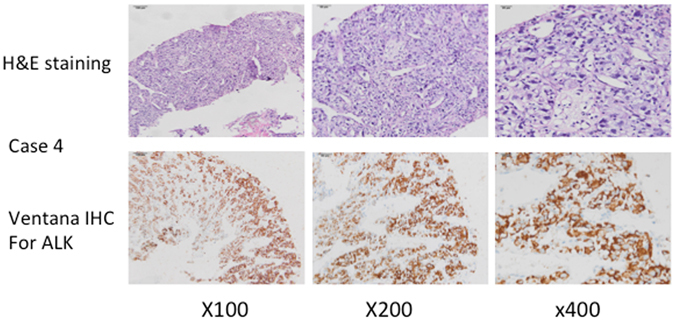
Immunohistochemical and H&E staining for EML4-ALK gene fusions. Case 4 harbored an EML4-ALK gene fusion that was discovered by NGS. Immunohistochemical staining with monoclonal rabbit antibody (Ventana D5F3, ROCHE, CH) confirmed the presence of an EML4-ALK fusion.

TP53 point mutations were found in 4 cases (57.1% of cases), followed in frequency by FAT3, MET, NCOR1, TSHR, APC, HGF, PIK3CA, EGFR, BRAF, BRIP1, and KMT2D, which all appeared in only 1 case (14.3% of cases) each (Fig. 3B). Four TP53 mutations were found on chromosome 17, but these mutations occurred in different exons: case 2 presented with a C to A mutation in exon 4, position 7577551; case 3 presented with a C to A mutation in exon 5, position 7578416; case 4 presented with a C to T mutation in exon 7, position 7577085; case 5 presented with an A to G mutation in exon 8, position 7579419 (Complementary Table 3). An EGFR exon 19 deletion was found in case 5 with an allele fraction of 0.5 on chromosome 7, position 55242464; this mutation changed the sequence AGGAATTAAGAGAAGC to A. An EML4-ALK gene fusion was found in case 4 with an allele fraction of 0.12 on chromosome 2 (Complementary Table 2). The mutation map of the EML4-ALK gene fusion is shown in Complementary Fig. 1. A BRAF.G469A mutation was detected in case 6 with an allele fraction of 0.24 on chromosome 7, position 140481402; this mutation resulted in the change of base C to G. A MET.S203F mutation was found in case 1 with an allele fraction of 0.16 on chromosome 7, position 116339746, exon 24; this mutation resulted in the change of base C to T. PIK3CA.H1047R and PIK3CA.H1048R mutations were found in case 3 with allele fractions of 0.33 and 0.34, respectively, on chromosome 3, positions 178952088 and 178952085; these mutations resulted in the change of base A to G. None of the TP53 point mutations appeared alone, as they were accompanied by other point mutations: in case 2, the TP53 mutation coincided with an HGF p. G520R mutation; in case 3, the TP53 mutation coincided with PIK3CA.H1047R and PIK3CA.H1048R mutations; in case 4, the TP53 mutation coincided with an EML4-ALK gene fusion; in case 5, the TP53 mutation coincided with an EGFR exon 19 deletion. As shown in Table 3, 2 of the 4 patients with TP53 mutations were males (50%, 2/4), and the other two were females. No significant associations were detected between the gene fusion mutation and the cancer stage, metastatic status, or sex. To confirm the NGS results, the ALK protein was evaluated using a monoclonal rabbit antibody (Ventana D5F3, ROCHE, CH) on a benchmark system. ALK was considered positive when at least 10% of the tumor cells stained with an intensity ≥2. The H2228 cell line was used as an external positive control. As shown in Fig. 4, ALK protein translocation was confirmed by Ventana IHC.

### Gene Mutations According to the Morphological Subtype of PSCs

As described above, pleomorphic carcinoma shows the highest incidence in PSCs and always shows pure malignant carcinoma components or NSCLC components (consisting of at least 10% malignant carcinoma components). We were unable to identify a correlation between genetic mutations and PSC subgroups. In our study cohort of 7 PSC cases, 5 cases had pure malignant carcinoma components, and the other 2 cases were of mixed type, containing adenocarcinoma components. An EGFR exon 19 deletion and a BRAF point mutation were both found in the mixed type. The mutation map of the EGFR exon 19 deletion is shown in Complementary Fig. 1. TP53 mutations were observed more frequently in pure PSCs (3 of 7 cases, 42.9%) than in mixed types (1 of 7 cases, 14.3%). Moreover, the other mutations were all found in the pure PSC subtype.

## Discussion

According to the WHO, poorly differentiated non-small-cell carcinomas of the lung with sarcoma-like differentiation (spindle and/or giant cell) or a sarcoma component (malignant bone, cartilage, or skeletal muscle) are unified within the umbrella term PSC^1^. Five subtypes are recognized: pleomorphic carcinoma, spindle cell carcinoma, giant cell carcinoma, carcinosarcoma, and pulmonary blastoma^1^. Although smoking plays a primary role in the etiology of PSC as in other lung cancers, some cases have been reported to be associated with asbestos exposure^12^. PSC has an average size of approximately 7 cm in diameter and most often presents as solitary masses in the upper lobes^13^. PSCs are highly aggressive and associate with a poorer prognosis than other lung carcinomas^14^. Fishback *et al*. reported that a tumor size >5 cm, a clinical stage >Stage I, and lymph node involvement are associated with shortened patient survival^15^. Linda W. Martin *et al*. retrospectively evaluated patients who underwent pulmonary resection for NSCLC during a 20-year period at the University of Texas MD Anderson Cancer Center and compared the recurrence and survival rates of patients with PSC in a cohort of patients with typical NSCLC. The 5-year survival rate for PSC patients was 24.5% compared with 46.3% for the NSCLC patients (p = 0.01). The median time to recurrence was 11.3 months and 61.4 months, respectively (p = 0.001)^16^.

The striking morphologic variability of the PSCs has important implications for diagnosis. Macropathologically, PSCs range from 2 cm to 18 cm in size and often invade the chest wall^15, 17^. Tumor consistency is often described as soft and fleshy to hard or rubbery. Sectional surfaces vary from white-gray to tan-yellow and frequently show areas of hemorrhage and necrosis and occasionally show cavitations^15, 17, 18^.

With small biopsies, the diagnosis of PSC may be difficult to substantiate because squamous cell carcinoma, adenocarcinoma, large cell carcinoma, and sarcoma elements are ambiguous by microscopy. Moreover, normal carcinoma and sarcoma components in these tumors are often inconspicuous. Therefore, in resection specimens, adequate sampling is essential and should include at least one section per centimeter of the maximum tumor diameter. This approach will generate a considerable number of histological slides for review because many PSCs are quite large. However, carcinosarcoma and pulmonary blastoma are rare compared with the other three types. In addition, giant cells and spindle cells always appear in the same microscopic field; thus, pleomorphic carcinoma is more commonly observed than spindle cell carcinoma and giant cell carcinoma^19^.

Morphologically, PSCs are highly variable, reflecting the histologic heterogeneity of lung cancers, which has important implications for diagnosis^20^. The giant cell-type of PSC is a carcinoma that only has anaplastic, giant tumor cells^1^. Malignant giant cells are discohesive; uninucleated or multinucleated; have moderate to abundant, dense, eosinophilic cytoplasm; often show emperipolesis by polymorphonuclear leukocytes or lymphocytes; and are typically anaplastic with many bizarre forms. Tumor cells are embedded in a fibrous or often myxoid stroma.

PSCs do not have specific radiologic characteristics, and thus, it is difficult to diagnose PSCs with only radiologic images, such as computerized tomography, magnetic resonance imaging, or positron emission tomography. Histology and immunohistochemistry are the gold standards for the diagnosis of PSC. Regarding immunohistochemistry, CEA, EMA, CK, CK7, chromogranin A, CD56, and synaptophysin can be used as markers for carcinomatous components, and desmin, vimentin, and smooth muscle/sarcomeric actin can be used as markers for sarcomatous elements^17, 21^.

For limited-stage PSCs, surgical resection is an effective treatment and provides adequate local control^22^. However, for metastatic PSC, platinum-based chemotherapy is commonly used for treatment as in other non-small cell lung cancer, although the efficacy is not ideal. A study by Vieira *et al*. included 97 patients with metastatic PSC, of whom 73% received platinum-based chemotherapy. Interestingly, the difference in PFS was not statistically significantly different between the patients that received or did not receive platinum-based chemotherapy, which was similar to the outcome observed in OS (7 months with platinum versus 5.3 months without, P = 0.096)^3^.

Because of the higher rate of resistance to conventional chemotherapy than other NSCLCs, more treatment options are needed for PSC patients, especially targeted drugs. Using NGS, followed by a careful ranking and validation strategy, we discovered a high gene mutation frequency in PSCs. In our cohort, all samples contained mutations in at least one of the 298-gene panel, and 85.7% of cases showed multiple mutations in a single gene (up to 5 different mutations). Our results are similar to those of studies by Fallet V. *et al*. and Filippo Lococo *et al*. In these two studies, NGS of 26 NSCLC-related genes was conducted to analyze the gene mutation status of PSCs. The authors reported that approximately 69% to 80% of PSC cases harbored at least one mutation and that PSCs show a higher mutation rate in the analyzed genes than the other NSCLCs^23, 24^. We discovered several mutation profiles for PSCs, including several known oncogenes and tumor suppressor genes (TSGs), such as TP53, EGFR, ALK, PIK3CA, BRAF, and MET, as well as five novel recurrent mutated genes, such as FAT3, NCOR1, TSHR, APC, and HGF. These results suggest that PSCs maintain a high degree of genetic instability for reasons that are currently unknown.

The protein coded by the TP53 gene resides in the nucleus and acts as a tumor suppressor through sequence-specific DNA binding to promote the repair of damaged DNA. Somatic mutations and the increased expression of TP53 are found in approximately 50% of NSCLCs^25, 26^. Przygodzki *et al*. found that TP53 point mutations appeared in approximately 27% of adenocarcinomas and in 43% of squamous cell carcinomas, with an incidence of 14% in pleomorphic carcinomas^13^. Their results showed that TP53 mutations in pleomorphic carcinomas often appear in exon 7 in squamous cell carcinomas and often in exon 8 in adenocarcinomas^13^. We discovered that 4 of the 7 patients (57.1%) harbored TP53 point mutations that occurred in distinct exons: exons 4, 5, 7, and 8. This result is similar to a study by Filippo Lococo *et al*., who observed that the TP53 gene was mutated in 55% of PSC cases and that the mutations were highly heterogeneous, with a high number of private mutations detected in only one or two cases. The authors suggested that these TP53 mutations are not the driver genes of PSC and that they could occur as a side effect of increased genetic instability^23^. However, the high incidence of TP53 mutations in human tumors and the fact that mutant TP53 often accumulates at high levels in tumor cells makes it a potential target for cancer therapy. Interestingly, we also identified 2 patients with combination mutations. One patient had EGFR and TP53 mutations, and the other had an EML4-ALK gene fusion and a TP53 mutation, which have rarely been described in PSC tumors^21^. Several studies have suggested that the reversion of functional TP53 can trigger tumor cell death and lead to tumor clearance, even if a tumor carries multiple genetic alterations that drive tumor growth^27, 28^. Generally, mutant TP53 tumors are less sensitive to conventional chemotherapy and have a worse prognosis than wild type TP53 tumors. The development of mutant TP53-reactivating drugs is expected to be an effective antineoplastic therapy. In future research, in addition to increasing sample sizes and data, the TP53 mutation status in PSC patients should be further evaluated.

Marked advances have been made in targeted molecular therapy for the treatment of molecularly defined subsets of NSCLC, and impressive efficacies have been achieved with EGFR and ALK tyrosine kinase inhibitors in EGFR-mutant and ALK gene fusion NSCLCs. However, the frequency of EGFR mutations in PSC is controversial. Leone *et al*. reported that approximately 9% of patients with PSC contained EGFR exon 19 deletions^29^, and Kaira K *et al*. reported a 20% incidence of EGFR mutations in Asian patients^8^. Two other studies did not find EGFR mutations in European patients with PSCs^7, 30^. However, PSC patients with EGFR mutations did not show satisfactory therapeutic effects to TKIs^31^. Therefore, because of the low incidence of mutations and the lack of clinical response, the clinical benefit from TKIs in PSC patients is uncertain. The EML4-ALK gene fusion incidence in Chinese NSCLC patients was approximately 3.77% (7/208), which consisted of an incidence of 15.2% (5/33)^32^ in female, non-smoking adenocarcinoma patients. These patients exhibit favorable sensitivities to ALK inhibitors, such as crizotinib. However, ALK inhibitors and BRAF inhibitors used for the treatment of PSC represent strategies of questionable efficacy owing to the lower frequencies of rearrangements and mutations in the EML4-ALK and BRAF genes reported previously in PSCs. In our study, we discovered one patient that harbored an EML4-ALK gene fusion by NGS, and we further confirmed the ALK protein fusion by Ventana IHC. Interestingly, this tumor was composed entirely of spindle cells and giant cells, with no obvious adenocarcinoma components. We will require more samples to evaluate the NGS data to determine the frequency of EML4-ALK gene fusions in PSC tumors and whether these EML4-ALK-positive patients are sensitive to ALK inhibitors. We also identified one patient with an EGFR 19 exon deletion and one patient with a BRAF point mutation. These 2 patients had pleomorphic carcinomas with at least 10% adenocarcinoma components, thus the point mutation may have occurred in the adenocarcinoma area. This result was similar to a study conducted by Fallet V. *et al*., who found EGFR mutations in 18 patients (22.2%), of whom only two exhibited EGFR-sensitizing mutations (2.5%) as identified in exon 21 (L858R). These were both found in male smokers with pleomorphic carcinomas harboring adenocarcinoma components^24^. These patients may benefit from EGFR TKIs and BRAF inhibitors like other NSCLCs, especially adenocarcinomas.

In 2015, Liu *et al*. identified MET mutations that caused exon 14 skipping in 8 of 36 (22%) patient cases^33^. The authors suggested that this type of MET mutation could be a driver gene and a potentially targetable event in PSC. In addition, in their cohort, a 74-year-old woman with stage IV PSC (widespread disease with lung, liver, and bulky mesenteric disease) was noted 3 months after resection of a tumor that harbored MET exon 14 skipping. Based on the presence of MET mutations, 250 mg of crizotinib was administered orally twice daily, which provided rapid and dramatic clinical improvement that was later confirmed radiographically as an excellent partial response using computed tomography scanning. However, in other studies, a significantly lower overall frequency of MET mutations was found^24, 34^. In our study, among the 7 cases, only case 1 harbored a MET SNP in exon 24. The patient died of her disease quickly after tumor resection and 4 cycles of postoperative chemotherapy; therefore, we were unable to test whether the patient could benefit from MET inhibitor therapy.

## Conclusions

Although our sample size was small because of limitations in the sample time requirements, our genetic analysis confirms that PSC harbors a high frequency of mutations in the *TP53* gene, similarly to some previous studies, suggesting that PSC is a distinct subtype of NSCLC. Furthermore, more functional studies should be conducted to identify new mechanisms and therapies for PSC patients with *TP53* point mutations.

### Consent

Written informed consent was obtained from the patients for the publication of this case report and the accompanying images. A copy of the consent form is available for review by the Editor-in-Chief of this journal.
